# The bottlenose dolphin (*Tursiops truncatus*): a novel model for studying healthy arterial aging

**DOI:** 10.1152/ajpheart.00464.2024

**Published:** 2024-07-26

**Authors:** Yara Bernaldo de Quirós, Sophia A. Mahoney, Nicholas S. VanDongen, Nathan T. Greenberg, Ravinandan Venkatasubramanian, Pedro Saavedra, Greg Bossart, Vienna E. Brunt, Zachary S. Clayton, Antonio Fernández, Douglas R. Seals

**Affiliations:** ^1^Department of Integrative Physiology, University of Colorado Boulder, Boulder, Colorado, United States; ^2^Institute of Animal Health and Food Safety, Universidad de Las Palmas de Gran Canaria, Las Palmas, Spain; ^3^Department of Mathematics, Universidad de Las Palmas de Gran Canaria, Las Palmas, Spain; ^4^Georgia Aquarium, Atlanta, Georgia, United States

**Keywords:** advancing age, cardiovascular disease, cetaceans, diving, endothelial function

## Abstract

Endothelial function declines with aging and independently predicts future cardiovascular disease (CVD) events. Diving also impairs endothelial function in humans. Yet, dolphins, being long-lived mammals adapted to diving, undergo repetitive cycles of tissue hypoxia-reoxygenation and disturbed shear stress without manifesting any apparent detrimental effects, as CVD is essentially nonexistent in these animals. Thus, dolphins may be a unique model of healthy arterial aging and may provide insights into strategies for clinical medicine. Emerging evidence shows that the circulating milieu (bioactive factors in the blood) is at least partially responsible for transducing reductions in age-related endothelial function. To assess whether dolphins have preserved endothelial function with aging because of a protected circulating milieu, we tested if the serum (pool of the circulating milieu) of bottlenose dolphins (*Tursiops truncatus*) induces the same arterial aging phenotype as the serum of age-equivalent humans. We incubated conduit arteries from young and old mice with dolphin and human serum and measured endothelial function ex vivo via endothelium-dependent dilation to acetylcholine. Although young arteries incubated with serum from midlife/older adult human serum had lower endothelial function, those incubated with dolphin serum consistently maintained high endothelial function regardless of the age of the donor. Thus, studying the arterial health of dolphins could lead to potential novel therapeutic strategies to improve age-related endothelial dysfunction in humans.

**NEW & NOTEWORTHY** We demonstrate that, unlike serum of midlife/older adult humans, age-matched dolphin serum elicits higher endothelial function ex vivo in young mouse carotid arteries, suggesting that the circulating milieu of bottlenose dolphins may be geroprotective. We propose that dolphins are a novel model to investigate potential novel therapeutic strategies to mitigate age-related endothelial dysfunction in humans.

## INTRODUCTION

Cardiovascular diseases (CVDs) are the leading cause of death globally, and advancing age is the primary nonmodifiable risk factor for CVD development (1). The world’s older population (≥65 yr of age) is rapidly increasing, with epidemiological models predicting a twofold increase by 2050 (2). With an increase in the proportion of the world’s older population, CVD prevalence is also expected to increase (1).

A key pathophysiological antecedent to CVD development is impaired conduit artery endothelium-dependent dilation (EDD), i.e., endothelial dysfunction (1). Independent of age, a reduction in endothelial function as measured by flow-mediated dilatation (FMD) via ultrasonography in the brachial artery before and after the dive has been observed after a single scuba dive (3), as well as after repetitive scuba (4) and breath-hold dives (5).

Cetaceans, an infraorder of marine mammals including dolphins and whales, have long lifespans (6) and have evolutionarily adapted to breath-hold diving for the purpose of feeding (7). To preserve oxygen during the dives, cetaceans leverage apnea, bradycardia, and peripheral vasoconstriction as a diving response (7). Peripheral vasoconstriction is key to reduce the oxygen consumption of tissues and organs that are less vital during the dive (i.e., kidneys) while maintaining normal blood pressure as well as blood flow to and oxygenation of essential organs (i.e., brain) (7). As a result, these animals experience repetitive cycles of peripheral tissue hypoxia-reoxygenation and disturbed shear stress that, in humans (8), induce endothelial dysfunction resulting in increased CVD risk. However, the prevalence of age-related CVD in cetaceans is negligible (9). Given that cetaceans have long lifespans that are relatively free of age-related CVD and dive continuously throughout their lives, with no apparent damage to their arteries, we propose that cetaceans may be a unique model of healthy arterial aging, potentially providing insights into strategies for clinical medicine.

Emerging evidence has shown that the circulating milieu (i.e., the bioactive factors in the blood) in humans changes with advancing age (10) and after diving (4, 11) and that it is at least partially responsible for transducing reductions in endothelial function with aging (12). As a first step toward testing our working hypothesis (i.e., cetaceans being a unique model of healthy arterial aging), we investigated if the circulating milieu of bottlenose dolphins (*Tursiops truncatus*), the most accessible and scientifically well-known cetacean species, preserves endothelial function of excised mouse carotid arteries, regardless of donor age. For this purpose, we incubated conduit (common carotid) arteries from young and old mice [i.e., a mammal species unrelated to dolphins and humans to prevent bias, and a well-established model of arterial aging (13)] with dolphin and human serum of equivalent ages) and measured endothelial function ex vivo via EDD to acetylcholine (ACh). Regardless of the age of the mouse donor, exposure of carotid arteries to serum from midlife/older (ML/O) adult humans resulted in lower endothelial function relative to exposure of carotid arteries to serum from age-equivalent dolphins.

## MATERIALS AND METHODS

### Mice Ethical Approval

Mouse procedures were reviewed and approved by the Institutional Animal Care and Use Committee at the University of Colorado Boulder (Protocol No. 2618). All procedures adhered to the guidelines set forth by the *Guide for the Care and Use of Laboratory Animals*.

### Mice Studies

Young (3–5 mo; *n* = 20: 6 female/14 male) and old (25 mo; *n* = 18: 6 female/12 male) C57BL/6N mice were obtained from the National Institute on Aging colony (maintained by Charles River). Mice of this strain and species are a well-established model of human arterial aging (13). Mice were allowed to acclimate to our conventional animal facilities for at least 2 wk prior to the study. Mice were group-housed by sex and maintained on a 12-h:12-h light/dark cycle. Mice were given ad libitum access to an irradiated pellet open formula (Teklad 7917; Envigo, Indianapolis, stored at room temperature) and drinking water (Boulder, CO, municipal tap water that underwent reverse osmosis and chlorination). Mice were euthanized via cardiac exsanguination under inhaled isoflurane anesthesia, and carotid arteries were immediately excised. Paired carotid arteries were then mounted in a culture pressure myograph system incorporating automated syringe drivers for intraluminal perfusion of sex-matched young or midlife/older dolphin serum for 24 h following our published protocol (12). As a control group, we used previously published responses from mouse carotid arteries incubated with healthy (nonobese, nonsmokers, and free of clinical disease) adult human serum of equivalent ages (young, 24 ± 1 yr of age; ML/O, 67 ± 3 yr of age) that were obtained in parallel in the same laboratory and at the same time (12). Serum human samples were obtained from participants of previous studies who gave written informed consent to perform follow-up analyses with samples collected during their visits. Serum samples were stored at −80°C. The Institutional Review Board of the University of Colorado Boulder approved all procedures.

In brief, carotid arteries were submerged within the culture myograph in a modified Krebs-buffered solution, which was continuously renewed via a peristaltic pump for nutrient replacement and maintained at 37°C. Concurrently, the diluted serum solution (5% serum in modified Krebs buffer) was perfused intraluminally in anterograde using a syringe driver. Carotid arteries were pressurized to 50–55 mmHg using a pneumatic pump emulating physiological conditions. At the end of the incubation period, the modified Krebs solution was replaced with a physiological salt solution, and vessels were preconstricted with 20 μM phenylephrine. EDD was assessed by measuring increases in the vessel diameter in response to increasing concentrations of ACh (1 × 10^−9^ to 1 × 10^−4^ M) added directly into the chamber. Two EDD dose responses were measured consecutively with a 30-min recovery in between. Following EDD, endothelium-independent dilation (EID) was assessed by measuring the increase in diameter in response to increasing concentrations of sodium nitroprusside (1 × 10^−9^ to 1 × 10^−3^ M), an exogenous nitric oxide (NO) donor. For more detailed information (e.g., equipment and reagent references and solution composition), please refer to our published protocol (12).

### Dolphin Serum

For age equivalency, the age and sex of 161 Sarasota Bay resident bottlenose dolphins spanning five generations were used. Dolphin serum from young (17.5 ± 8 yr of age; *n* = 16, 5 female/11 male) and ML/O (28.5 ± 3 yr old, *n* = 4: 1 female/3 male) adults was obtained from archived samples at the National Marine Mammal Tissue Bank and used under permit No. 24350 issued by the National Marine Fisheries Service. These samples were collected during health-capture assessments of free-ranging dolphins and stored at −80°C. Only serum samples from dolphins of known age were used (Table 1). Although the National Marine Mammal Tissue Bank has a large collection of samples, the age of the dolphins is known only for a small fraction of animals, and older animal samples are more scarce. Adult females with calves are not captured to prevent stress on the calf. Hence, the availability of adult female samples of known age is very limited. Adolescent (sexually mature) and young adult dolphins were merged, as no differences were observed between these groups. Dolphin and human serum samples were heated to 56°C for 30 min to enable cross-species compatibility and diluted to 5% in a modified Krebs-buffered solution (12).

**Table 1.** Dolphin sample size, sex, and ages

	Adolescents	Young Adults	ML/O Adults
*N* (females/males)	8 [3/5]	8 [2/6]	4 [1/3]
Age, yr	13 [12–14]	21 [21–21.3]	28.5 [27–30]

Values are medians [interquartile ranges, 25th–75th percentile]; *N*, number of dolphins. ML/O, midlife/older.

### Statistics

The minimum sample size was estimated with G*Power 3.1 software using peak EDD data of young and old mouse carotid arteries after incubation with a pool of young and old mouse serum, respectively (12), with an α-error probability of 0.05 and 0.95 power. Data were summarized using the median and interquartile range [25th–75th percentiles]. The medians of peak EDD, area under the curve (AUC), and ACh concentration that provoked a 50% dilation (logEC_50_) for the four groups (young and ML/O human serum and young and ML/O dolphin serum) were compared using the Kruskal–Wallis test. If statistical significance was found, multiple comparisons were performed using Conover’s all-pairs rank comparison test for medians (14). Data were analyzed using the R package, version 4.2.1 (R Development Core Team, 2022) (15). In all instances, statistical significance was set at *P* < 0.05. GraphPad Prism version 10.2.2 was used for graphical purposes.

## RESULTS

To test the effects of the circulating milieu on age-related endothelial function, we measured EDD and EID in young and old mouse carotid arteries exposed to young and ML/O serum from humans and dolphins: four exposure groups in total.

**Figure 1.**
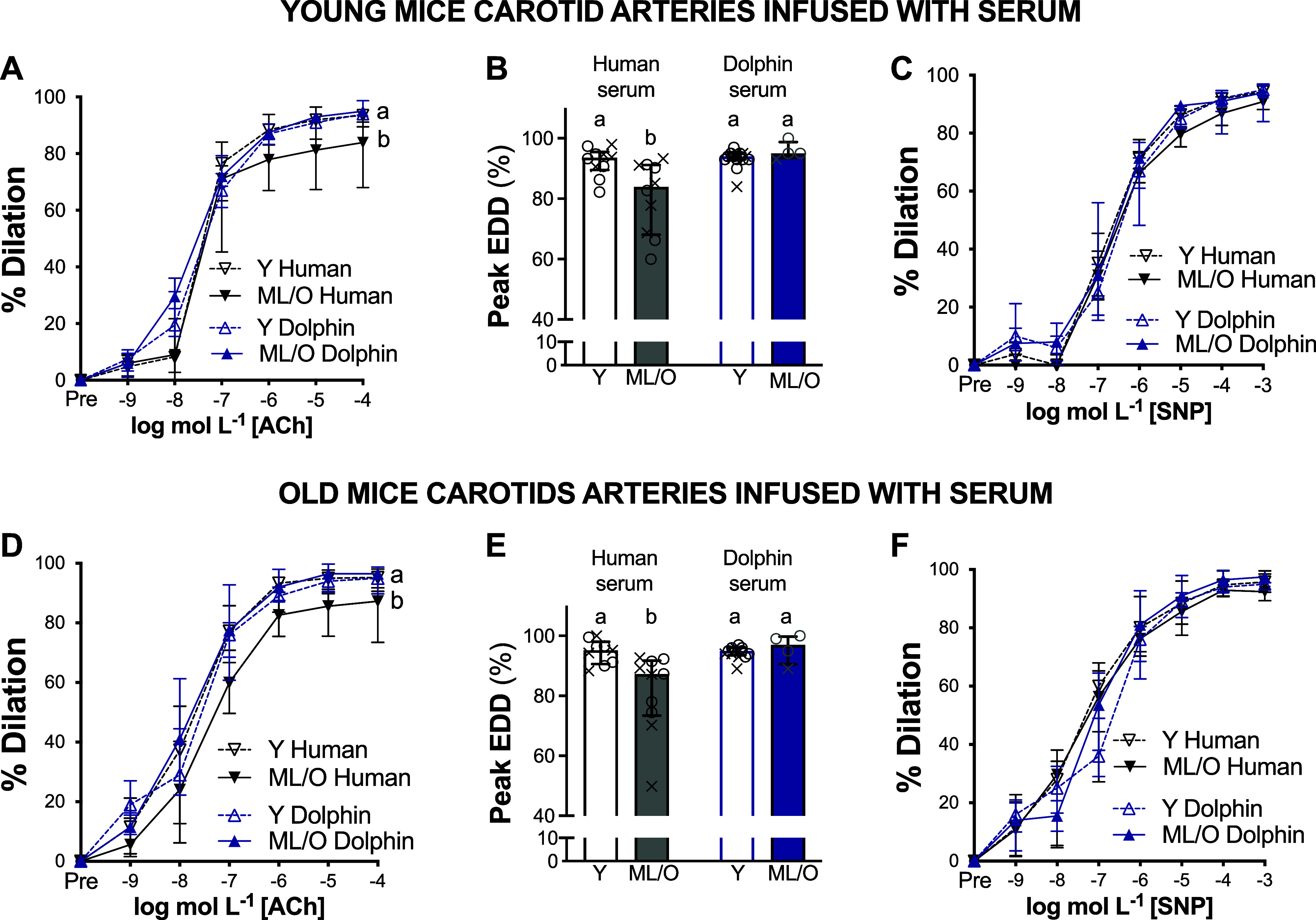
Ex vivo measures of endothelial function. Endothelium-dependent dilation (EDD; *A* and *B*) and endothelium-independent dilation (EID; *C*) of young mice carotid arteries, as well as EDD (*D* and *E*) and EID (*F*) of old-mice carotid arteries following exposure to the same-serum groups: *n* = 10 (5 females/5 males) young adult humans (Y Human), *n* = 10 (5 females/5 males) midlife/older adult humans (ML/O Human), *n* = 16 (5 females/11 males) young adult dolphins (Y Dolphin), *n* = 4 (1 female/3 males) midlife/older adult dolphins (ML/O Dolphin). ×, female data; ○, male data. Data represent medians and interquartile ranges. ^a,b^*P* < 0.05, significant differences.

We found similar results in young and old mouse carotid arteries. No significant differences among the four groups were found for AUC or logEC_50_ (Table 2). Peak EDD differed across groups (*P* = 0.002 in young and *P* = 0.001 in old arteries) (Fig. 1, *A*–*C*), but no significant differences were found for peak EID (*P* = 0.780 in young and *P* = 0.198 in old arteries) (Fig. 1, *D*–*F*).

**Table 2.** AUC and concentration of ACh that provokes a 50% dilation (logEC_50_) for EDD

	Serum Exposure				Kruskal–Wallis Test
	Young human	ML/O human	Young dolphin	ML/O dolphin	*P* value
*N*	10	10	16	4	
AUC	301 [287–362]	278 [232–323]	322 [304–335]	333 [330–350]	0.080
logEC_50_	−7.2 [−7.7 to −7.0]	−7.1 [−7.4 to −6.3]	−7.3 [−7.5 to −7.2]	−7.5 [−7.7 to −7.4]	0.215

Data were summarized using the medians and interquartile range (IQR = 25th−75th percentile); *N*, number of human and dolphin subjects, respectively. ML/O, midlife/older. Area under the curve (AUC) and concentration of acetylcholine (ACh) provoke a 50% dilation (logEC_50_) for endothelium-dependent dilation (EDD).

In young mouse carotid arteries exposed to young human serum (young control group), the peak EDD was 93.5% (90.6–94.8%). Peak EDD was lower after incubation of young carotid arteries with ML/O human serum (85.1% [77.8–91.1%], *P* = 0.022 vs. young control group) (Fig. 1, *A* and *B*, gray). In contrast, peak EDD was not different than young control group values after incubation with young (93.8% [92.7–94.7%], *P* = 0.974) or ML/O (95.1% [94.4–96.5%], *P* = 0.293) adult dolphin serum (Fig. 1, *A* and *B*, blue).

In old mouse carotid arteries exposed to ML/O adult human serum (old control group), the peak EDD was 87.2% (75.4–90.9%). Peak EDD was higher after incubation of old carotid arteries with young human serum (95.2% [91.2–96.6%], *P* = 0.002 vs. old control group) (Fig. 1, *C* and *D*, gray), as well as with young (94.8% [94.4–95.7%], *P* < 0.001) and ML/O (97.2% [93.0–99.3%], *P* = 0.003) adult dolphin serum (Fig. 1, *C* and *D*, blue).

In both young and old mouse carotid arteries, peak EDD was lower after incubation with ML/O adult human serum compared with peak EDD levels after exposure to young adult human serum. However, there were no differences in EID (Fig. 1, *D* and *F*), indicating that the lower peak EDD after exposure to ML/O adult human serum occurred in an endothelium-specific manner (12).

Overall, these results suggest that, unlike the circulating milieu of humans that impairs endothelial function with aging, the circulating milieu of dolphins preserves endothelial function regardless of the age of the dolphins.

## DISCUSSION

To our knowledge, there are yet no in vivo measures of endothelial function in dolphins. As a first approach, this study focuses on the effect of the circulating milieu of bottlenose dolphins on the endothelial function of mouse arteries ex vivo. Our results provide the first ancillary insight into what may be expected in dolphins.

Endothelial function of carotid arteries isolated from both young and old mice was lower after incubation with ML/O adult human serum. In humans, NO bioavailability decreases with advancing age, in part because of an increase in tonic reactive oxygen species (ROS)-mediated oxidative stress and a subsequent increase in the production of proinflammatory cytokines (1). In addition, reperfusion of tissues after ischemia and diving increases ROS and inflammation independent of age, changes that are reflected in the circulating milieu (4, 11). Moreover, we have shown in a recent article, a strong correlation between the ex vivo measures of mouse carotid artery EDD to ACh incubated with human serum and the in vivo FMD of the human donor (12). Interestingly, the peak EDD of old mouse arteries incubated with ML/O adult dolphin serum was as high as that of young mouse arteries incubated with young adult dolphin and human serum. Hence, ML/O dolphin serum did not have the same unfavorable effects on carotid arteries ex vivo. Unlike the circulating milieu of ML/O humans, it did not induce a state of lower endothelial function.

Circulating factors may be lost or altered during storage (−80°C) or heating to inactivate the complement. However, human and dolphin serum were treated the same way, and differences were still only found for ML/O human serum.

Cetaceans include some of the longest-living mammal species. For example, the Bowhead whale (*Balaena mysticetus*) is the longest-lived species at an astounding 211 yr (6). They live a fully aquatic lifestyle characterized by continuous diving throughout their life, although diving abilities vary among cetacean species. The genetics of these whales have been studied to better understand the mechanisms underlying their longevity (16, 17). Few have studied their resistance to hypoxia and reoxygenation insults that they experience during dives with no apparent damage (18, 19). Current literature indicates that cetaceans produce excessive ROS after blood flow restriction (hypoxia) and reperfusion, similar to terrestrial mammals (18, 19). Deep- and long-diving cetaceans produce higher ROS than shallow- and short-diving cetaceans (18). However, deep-long diving cetaceans have higher antioxidant capacities (18). Indeed, at least under cell culture conditions, the higher antioxidant defenses of bottlenose dolphins resulted in an attenuated inflammatory response to ROS (19). Seals, a different group of diving mammals that presents convergent evolution with cetaceans, maintain their antioxidant activity with advancing age (20). Cetaceans likely also maintain their antioxidant capacity into late life, although further research is needed to confirm this hypothesis.

It has been reported for different laboratory animals that cardiac ischemic preconditioning (i.e., short cycles of ischemia/reperfusion) protects vascular endothelial function (21). In addition, remote ischemic preconditioning releases cardioprotective humoral factors (22). Repeated cycles of hypoxia/reoxygenation of peripheral tissues in cetaceans, similar to the ischemia/reperfusion cycles during ischemic preconditioning, may lead to a release of beneficial humoral factors that may protect vascular endothelial function.

Unlike seals, cetaceans are fully aquatic animals and, hence, are more challenging to study. Their genetics might provide mechanistic insight that would otherwise be markedly difficult to test in cetaceans. Indeed, cetaceans have a positive selection of antioxidant and anti-inflammatory-related genes suggesting an enhanced protective stress response in cetaceans (23). This is likely reflected in the circulating milieu, which might protect the arteries from the insults of diving and aging, given their similarities. Although further research in the composition of the circulating milieu is necessary to confirm this hypothesis. In addition, whales express high amounts of the argininosuccinate lyase (*Asl*) gene (17), which is essential for both arginine synthesis and NO production (24). Given the existing literature and our results, further research is warranted to explore the role of NO bioavailability in mediating this preservation.

Our study represents the first investigation of arterial aging in cetaceans. Bottlenose dolphins are not the longest lived or the best divers, but they are the most highly characterized cetacean species, regarding life history, anatomy, physiology, and health population status (25, 26). Access to this information has enabled us to extrapolate age equivalencies between dolphin and human chronological age using the age and sex population structure of the resident Sarasota Bay bottlenose dolphin community with 161 individuals studied and spanning five generations (27). Maximum human lifespan is ∼115 yr of age (28). The US National Institute of Health (NIH) considers ML to be 45–64 yr of age (29) and older adults as people aged 65 yr or older (30). The maximum known lifespan for a female bottlenose dolphin is 59 yr of age, whereas males do not surpass 50 yr of age. Chronologically, dolphins aged 27–30 yr may be considered ML/O adults.

The bottlenose dolphin is the cetacean species with the most serum collected for health assessments given that it is a coastal species that is relatively easy to capture with minimal invasiveness or harm (26). Although the number of ML/O dolphin serum samples of known age available in the biobank was small, this limitation was overcome by the small dispersion of the data. In the future, more samples, including samples from older dolphins may become available. In addition, dolphins kept in captivity could provide insight into whether the properties of the circulating milieu of dolphins is a phylogenetic adaptation that evolved over millions of years or if, instead, it is an acclimatization to diving, since dolphins in captivity do not dive as deep or as long as their free-range counterparts. Future studies are needed to investigate the factors within the circulating milieu involved in protecting dolphins from developing endothelial dysfunction despite aging and diving habits.

### Conclusions

In summary, mounting evidence suggests that cetaceans, long-lived mammals that dive continuously, show no signs of associated tissue or arterial damage. In this study, we demonstrate that the circulating milieu of bottlenose dolphins is geroprotective ex vivo in mouse carotid arteries. Taken together, the diving adaptations of cetaceans may protect their endothelium from typical age-related insults of terrestrial mammals. Hence, cetaceans could serve as a model to investigate targets, mechanisms, and potential therapies for preventing and/or treating adverse arterial aging and promoting cardiovascular health and longevity in humans.

## DATA AVAILABILITY

Data will be made available upon reasonable request.

## GRANTS

This project has received funding from European Union’s Horizon 2020 Research and Innovation Programme’s Marie Sklodowska-Curie Grant MSCA-IF-2019: 892267; National Institutes of Health (NIH) Exploratory/Developmental Research Grant R21AG078408 (to D.R.S); and NIH Grants F31HL165885 (to S.A.M), F31HL164004 (to N.T.G.), K99/R00HL151818 (to V.E.B), and K99/R00HL159241 (to Z.S.C.). Pressure myography equipment was purchased using funds from Colorado Clinical and Translational Sciences Institute Grant CO-M-20-024 (to V.E.B) and Department of Integrative Physiology discretional funds (to D.R.S.).

## DISCLOSURES

No conflicts of interest, financial or otherwise, are declared by the authors.

## AUTHOR CONTRIBUTIONS

Y.B.d.Q., S.A.M., V.E.B., Z.S.C., A.F., and D.R.S. conceived and designed research; Y.B.d.Q., S.A.M., N.S.V., N.T.G., R.V., G.B., and Z.S.C. performed experiments; Y.B.d.Q. and P.S. analyzed data; Y.B.d.Q., S.A.M., N.T.G., V.E.B., Z.S.C., and D.R.S. interpreted results of experiments; Y.B.d.Q. prepared figures; Y.B.d.Q. drafted manuscript; Y.B.d.Q., S.A.M., N.S.V., N.T.G., R.V., P.S., V.E.B., Z.S.C., A.F., and D.R.S. edited and revised manuscript; Y.B.d.Q., S.A.M., N.S.V., N.T.G., R.V., P.S., G.B., V.E.B., Z.S.C., A.F., and D.R.S. approved final version of manuscript.
